# Study on analytical characteristics of *Nicotiana tabacum* L., cv. *Solaris* biomass for potential uses in nutrition and biomethane production

**DOI:** 10.1038/s41598-019-53237-8

**Published:** 2019-11-14

**Authors:** Antonella Fatica, Francesco Di Lucia, Stefano Marino, Arturo Alvino, Massimo Zuin, Hayo De Feijter, Boudewijn Brandt, Sergio Tommasini, Francesco Fantuz, Elisabetta Salimei

**Affiliations:** 1 Dipartimento Agricoltura, Ambiente, Alimenti, Università degli Studi del Molise, via Francesco De Sanctis, 1, Campobasso, 86100 Italy; 2 Sunchem BV, Amsterdam, The Netherlands; 3 Agronomist Consultant, Pordenone, Italy; 4 Scuola di Bioscienze e Medicina Veterinaria, Università degli Studi di Camerino, via Gentile III Da Varano, 62032 Camerino, Italy

**Keywords:** Field trials, Biofuels

## Abstract

In order to limit the smoking tobacco sector crisis, a new non-GMO *Nicotiana tabacum* L. cv. *Solaris* was proposed as oil seed crop. Residues of oil extraction were successfully used in swine nutrition. The aim of this study was to explore the full potential of this innovative tobacco cultivar as multitasking feedstock non interfering with the food chain. In the triennium 2016–2018, samples from whole plant, inflorescence and stem-leaf biomass were collected in three experimental sites and analysed for chemical constituents, including fibre fractions, sugars and starch, macro-minerals and total alkaloids. The KOH soluble protein content and the amino-acid profile were also investigated as well as the biochemical methane potential. All the analyses were performed according to official methods and results were compared with values reported in literature for conventional lignocellulosic crops and agro-industry residues. The average protein content, ranging from 16.01 to 18.98 g 100 g^−1^ dry matter respectively for stem-leaf and whole plant samples, and their amino-acid profile are consistent with values reported for standard grass plant. These findings suggest the potential use of cv. *Solaris* in industrial food formulations. Moreover, considering the average content of both fibre available for fermentations (72.6% of Neutral Detergent Fibre) and oils and fats (7.92 g 100 g^−1^ dry matter), the whole plant biomass of cv. *Solaris* showed good attitude to anaerobic fermentation, confirmed by the biochemical methane potential of whole plant (168 Nm^3^ t^−1^ organic matter). Similarly, results allow to define the cv. *Solaris* biomass as a good quality forage apt to ensiling for its chemical composition. The low total alkaloids content of cv. *Solaris*, in average 0.3 g 100 g^−1^ dry matter, was previously reported not to affect growth performances and welfare traits of dairy heifers. These are the first results showing the multitasking potential use of cv. *Solaris* biomass, that could allow the recovery of tobacco cultivation know-how especially in marginal areas.

## Introduction

The European *Nicotiana tabacum* L. cultivation has decreased considerably in recent years because restriction of subsidies^1^ and smoking tobacco production is reported to account for about 219,243 tons^2^.

Italy is the largest tobacco producer in the European Union, with an amount of 21% of the total European tobacco production^2^. However, in Italy the crisis of the sector is particularly serious, with a reduction of both the production (from 110,000 tons in the 2007 to 46,060 in the 2017) and the cultivated areas, reduced from 32,000 hectares in 2007 to 14,548 hectares in 2017^2^. Tobacco cultivation in Italy is concentrated in Campania, Umbria, Veneto and Toscana regions, where a strong cultivation know-how is available in terms of both technique and agricultural machinery.

In order to limit the crisis affecting the tobacco sector in Italy, thanks to 15 years of research, a new cultivar of *Nicotiana tabacum* L. was developed for maximizing the production of seeds with high content of oil used as biofuel or biomass source^1^. This cultivar, named *Solaris*, is a non-GMO developed and patented as “energy tobacco” (PCT/IB/2007/053412)^3,4^, registered in 119 countries and granted in over 75 countries, including USA, all Africa, EuroAsia, Italy, Russia and Australia.

The genetic improvement was addressed to a sustainable industrial “no food” culture, having no competition with plants produced as food for humans or animals.

**Figure 1:**
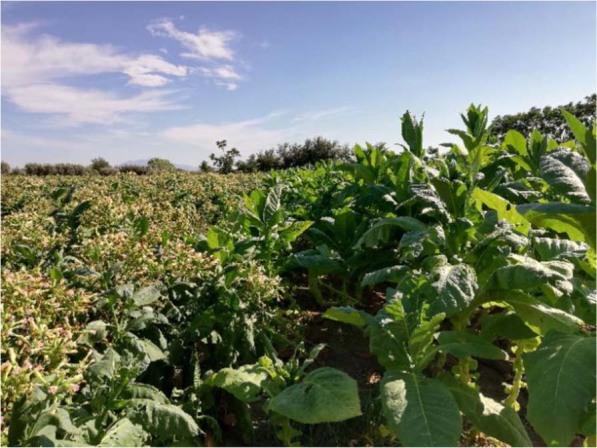
*Nicotiana tabacum* L. cv. *Solaris* (left) with reduced size and elevated number of flowers compared to traditional cultivar of smoking tobacco (right).

*Nicotiana tabacum* L. cv. *Solaris* compared with smoking tobacco varieties contains low levels of nicotine and maximizes the production of flowers/seeds, reducing leaf growth (Fig. 1).

The industrial uses of *Nicotiana tabacum* L. cv. *Solaris* as energy biomass were firstly reported by Grisan and colleagues^1^ that tested the tobacco *Solaris* cultivation as sustainable bioenergy chain in North and Central Italy. The cold-press extraction from seeds showed that the content of oil, used as jet biofuel, in tobacco seeds is around 38–40% and the oil cake coproduct may be useful as a high protein and energy feed for livestock^4^. So, given the chemical and nutritional characteristics, the cake obtained from the extraction of oil from tobacco seeds, was successfully introduced in piglets’ diet^5^.

The strategy proposed by Grisan and colleagues^1^ included two seed harvests: the first plant apexes collections carried out from the end of July to the first decade of August (80–90 days after transplanting), and the second seed harvest carried out in the second half of October (140–150 days after transplanting) when, on the other hand, the environmental conditions can be critical.

Consistently with its strong vegetative capacity and its short cultivation cycle (80–90 days the first harvest)^1^, the *Solaris* tobacco biomass has been here investigated following an alternative strategy, *i.e*. 50–60 days after the first collection of the plant apexes (seeds), the total biomass harvesting may occur within the same vegetative season. The large amounts of biomass harvested after the first seed collection could be used in other sectors, such as for biomethane production^6^, animal nutrition or food industry, consistently with a circular supply chain (circular economy) approach.

The aspects related to alternative uses of biomass from *Nicotiana tabacum* cv. *Solaris* are at the moment unknown, therefore, aiming to contribute to the advances of knowledge on this innovative culture and its potentialities, the goal of the present trial was to study the chemical characteristics, including amino acid profile and mineral content, and the potential biomethane production of cv. *Solaris* biomass.

Results were then compared with literature data on conventional plant materials used as source of biomass for nutrition, *i.e*. animal feeding and food supplement, and biomethane production.

## Materials and Methods

The study on *Nicotiana tabacum* L. cv. *Solaris* biomass has been carried out during the three-year period 2016–2018 in three sites located in Vicenza (45°17′25.4″N 11°30′09″E), Chieti (42°10′0″N 14°20′0″E) and Perugia (42°59′0″N 12°25′0″E) provinces, adopting conventional farming techniques of the areas^1^.

The scalar collections of fifteen cv. *Solaris* tobacco samples were made in years 2016, 2017, 2018 for Vicenza province, in 2017 for Chieti province and in 2018 for Perugia province.

**Figure 2:**
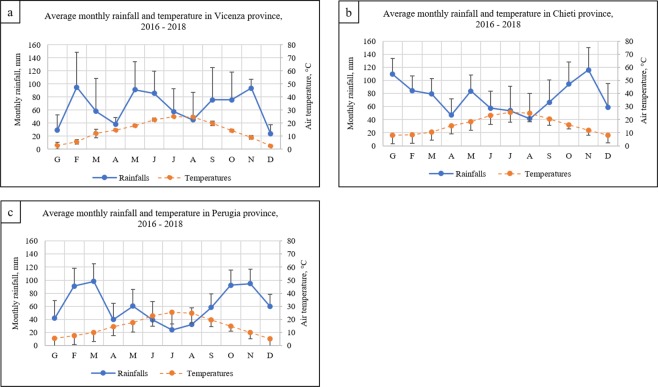
Thermal-pluvio-metric diagrams for 2016, 2017 and 2018 about Vicenza^7^ (**a**), Chieti^8^ (**b**) and Perugia^9^ (**c**) province.

Data on thermal-pluvio-metric conditions were collected by weather stations located in Vicenza, Chieti and Perugia provinces, near the study areas. Data were provided by ARPA Veneto^7^, HORT@ Agrometeorological Network^8^ and Regional Hydrographic Service^9^, respectively for Vicenza, Chieti and Perugia province. As depicted in Fig. 2(a–c), in the three years study the average temperature ranged monthly from 13.6 °C to 26.1 °C during the cultivation period (May–October), when total rainfalls ranged from 2.8 to 140.2 mm.

The field experiment was carried out in each year and site in randomized blocks with three replications. Each plot was 50 m^2^ for a total surface of 600 m^2^. Tobacco plants were transplanted after tillage carried out by ploughing (moldboard plow - depth of 50 cm) and followed by secondary harrowing in order to reduce the clod size. Nitrogen, phosphorus and potassium were distributed based on the soil analysis, in total were distributed 120 kg of N, 60 kg of P_2_O_5_, 70 kg of K_2_O by fertigation system. Insecticide products (z-cypermethrin) were buried on the ground to prevent attacks of terricolous cutworms in the transplantation stage. During the season, fungicide treatments (cooper and metalaxil) and insecticide (indoxacarb) treatments were carried out to protect the maturing capsules from fungal disease and bollworm.

In order to compare the effect of plant density, the transplant was performed mechanically on May, with two transplanting densities, *i.e*. 3.6 and 4.8 plants per m^2^. The plants were arranged in rows 90 cm apart and the distance between the plants on the row was 31 cm and 23 cm, respectively for the 3.6 and 4.8 plants per m^2^ density.

The herbicide treatment was performed the day after the transplant using a clomazone based product.

Drip irrigation (about 180 m^3^ ha^−1^ per watering) was adopted and the total amount ranged from about 1000 m^3^ ha^−1^ (6 waterings) to about 1450 m^3^ ha^−1^ (8 waterings), according to weather conditions and practices in use by local farmers.

The seed harvest was carried out manually by cutting the plant apexes (mature capsules) from the end of July to the first decade of August (80–90 days after transplanting). The seeds collected were weighted and seed yield recorded.

The total biomass was then harvested between the months of September and October (130–150 days after transplanting), depending on altitude, rainfall and temperature. The green biomass of cv. *Solaris* harvested by a common shredding machine for biomass collection, was weighted. Total biomass yield was recorded and sampled.

Biomass samples were collected as fractions of whole plant (1), inflorescence (2, flowering apex), stem-leaf (3) for years 2016 and 2017, while in 2018 only the whole plant was sampled. All samples underwent natural drying and chopping (4 cm).

Chemical analyses of all samples were carried out by an accredited laboratory (Mérieux NutriSciences Corporation, Resana, TV, Italy) according to official methods. The analyses, performed in three replicates, were focussed on the typical constituents (Humidity, Crude Protein, Crude oils and fats, Crude ash)^10^, plus fibrous components, including Neutral Detergent Fibre (NDF), Acid Detergent Fibre (ADF) and Acid Detergent Lignin (ADL)^11,12^, for all samples. The content of sugars (glucose, fructose, sucrose)^11,12^, starch^10^ and macro-minerals (Ca, P, Mg, K, Na and chlorides)^10–12^ were investigated on samples from 2016. On the same samples, the nitrogenous fraction was further investigated by the analyses of KOH-soluble proteins and amino acids (Asp, Thr, Ser, Glu, Pro, Gly, Ala, Val, Ile, Leu, Tyr, Phe, Lys, His, Arg) after hydrolysis^10^. Besides that, for a more complete scenario on the analytical characteristics of the biomass, consistent with the hypothesis of the destination of cv. *Solaris* in anaerobic digestors or ruminant nutrition, data on fibre fractions were subjected to further evaluations, *i.e*. hemicellulose = NDF-ADF, cellulose = ADF-ADL, available fibre = NDF-ADL^13^. Moreover, the content of Non-Fibrous Carbohydrates has been calculated as the difference to 100 of the sum of the crude protein, crude oil and fat, crude ash and NDF contents, expressed on a dry matter basis^13^. Consistently with the hypothesis of cv. *Solaris* whole plant used as forage, the determination of total alkaloids was also carried out (UNI EN ISO 2881/1992) and results have been expressed as nicotine. As reported in literature, nicotine is in fact the principal tobacco alkaloid accounting for about 95% of the total alkaloid content^14^.

As related to cv. *Solaris* attitude to anaerobic fermentation, the biochemical methane potential (BMP) has been tested on whole plant samples from 2017 and 2018 by a certified laboratory (CRPA LAB, Reggio Emilia, Italy), according to anaerobic digestion batch procedure (UNI EN ISO 11734/2004).

To evaluate the effects of density, all data were processed by analysis of variance (IBM SPSS, ver. 25). Seed and biomass production as well as the investigated analytical characteristics were not significantly affected by the two densities that were not further considered in this paper. Results are reported as mean ± standard deviation (s.d.), on dry matter (d.m.) basis except for BMP results reported on organic matter (o.m.) basis and are compared with values reported in literature for grass and legume plants.

Association between thermo-pluvio-metric data observed in the cultivation period, *i.e*. May-October, and organic matter, crude protein, crude oil and fat, crude ash, NDF, ADF and ADL contents of whole plant cv. *Solaris* biomass, was examined by two tailed Pearson correlation test (IBM SPSS, ver. 25).

## Results and Discussion

### Biomass yield and basic chemical characteristics

Table 1 shows for the first time that the yield of total biomass averaged 70.82 t ha^−1^ per year, ranging from 65.0 to 76.6 t ha^−1^. The seed yield averaged per year 2.1 (±0.16) t ha^−1^, confirming the data observed by Grisan and colleagues^1^ in Northern and Centre Italy. The mean temperature values observed in the three years study during the cultivation period was significantly related to the biomass yield (r = 0.925, P = 0.001) but not with rainfalls, as expected effect of waterings.

**Table 1:** *Nicotiana tabacum* L. cv. *Solaris* biomass produced per year in the study.

	Biomass
Mean, t ha^−1^	70.82
s.d., t ha^−1^	5.73
Min, t ha^−1^	65.00
Max, t ha^−1^	76.58

s.d. = standard deviation.

Biomass samples were characterized by a low dry matter content, *i.e*. 19.32 (±3.78) g/100 g. The organic matter content of whole plant fraction was in average 82.80 ± 2.36 g/100 g dry matter (d.m.), ranging from 80.09 (±3.83) g/100 g d.m. and 85.69 (±2.37) g/100 g d.m. in stem-leaf and inflorescence fraction, respectively (Table 2).

**Table 2:** Average content (±s.d.) of chemical components and biochemical methane potential of cv. *Solaris*. Values reported in literature for grass and legume plants are shown for comparison^15–17,27,28^.

	Unit	cv. *Solaris* Whole plant	cv. *Solaris* Inflorescence	cv. *Solaris* Stem-Leaf	Grass plant	Legume plant
Organic matter	g 100 g^−1^ d.m.	82.80 (2.36)	85.69 (2.37)	80.09 (3.83)	90.75 (3.04)	89.56 (1.92)
C. Protein	g 100 g^−1^ d.m.	18.98 (2.36)	24.85 (1.96)	16.01 (2.64)	11.41 (3.58)	19.00 (2.73)
Sol. Protein	g 100 g^−1^ d.m.	6.83 (1.04)	10.97 (1.08)	4.93 (0.91)	n.a.	n.a.
C. Oil and Fat	g 100 g^−1^ d.m.	7.92 (4.06)	14.16 (6.99)	3.06 (1.53)	2.88 (0.50)	2.64 (0.57)
C. Ash	g 100 g^−1^ d.m.	17.20 (2.36)	14.31 (2.37)	19.91 (3.83)	9.25 (3.04)	10.44 (1.92)
NDF	g 100 g^−1^ d.m.	46.64 (4.07)	45.97 (10.77)	47.65 (6.96)	58.88 (8.01)	49.27 (6.51)
ADF	g 100 g^−1^ d.m.	37.49 (4.99)	37.48 (8.67)	36.26 (7.49)	33.52 (7.67)	34.31 (6.23)
ADL	g 100 g^−1^ d.m.	12.87 (3.87)	17.72 (7.11)	8.14 (2.57)	5.68 (2.69)	7.59 (1.65)
Available. Fibre	g 100 g^−1^ d.m.	33.77 (4.14)	28.24 (5.85)	39.51 (4.80)	53.20 (6.55)	41.68 (6.87)
Hemicellulose	g 100 g^−1^ d.m.	9.14 (2.07)	8.48 (4.28)	11.39 (2.73)	22.11 (3.70)	14.52 (8.05)
Cellulose	g 100 g^−1^ d.m.	24.62 (4.31)	19.76 (2.88)	28.12 (5.02)	30.82 (5.65)	27.16 (5.38)
Starch	g 100 g^−1^ d.m.	1.75 (073)	2.32 (1.77)	1.67 (0.75)	24.19 (6.04)	n.a
Sugar	g 100 g^−1^ d.m.	0.88 (0.56)	1.41 (0.87)	0.42 (0.26)	8.41 (7.65)	1.26 (0.96)
Nicotine	g 100 g^−1^ d.m.	0.30 (0.20)	n.a.	n.a.	n.a.	n.a.
Biomethane	Nm^3^ t^−1^ o.m.	168.0 (47.0)	n.a.	n.a.	135.0 (8.7)	202.5 (53.0)

C. Protein = crude proteins; Sol. Protein = KOH Soluble Proteins; C. Oil and Fat = Crude oils and fats; C. Ash = Crude ash; NDF = Neutral Detergent Fibre; ADF = Acid Detergent Fibre; ADL = Lignin Acid Detergent; Nicotine = total alkaloids as nicotine; d.m = dry matter; o.m = organic matter; n.a. = not available.

The crude protein content of the inflorescence portion (24.85 ± 1.96 g/100 g d.m.) is in line with values reported in literature for the seed cake^5^, while the average values detected for whole plant and stem-leaf biomass samples (Table 2) lie at the level of values reported for the common sainfoin (*Onobrychis sativa* Lam.) and Italian sainfoin (*Hedysarum coronarium* L.) among the legume plants and for Italian ryegrass (*Lolium italicum/multiflorum* Lam.), cocksfoot (*Dactylis glomerata* L.) and sorghum (*Sorghum vulgare* L.) as grass plants^13,15–17^. Lower crude protein contents are reported for grass plants, such as triticale (*x Triticosecale*), barley (*Hordeum vulgare* L.) and corn (*Zea mays* L.), while higher values are found in legumes such as alfalfa (*Medicago sativa* L.), vetch (*Vicia sativa* L.), white clover (*Trifolium repens* L.) and red clover (*Trifolium pratense* L.)^15–18^. Among the raw materials with high fibre content, the same protein levels of whole plant and stem-leaf are intermediate between the protein content of alfalfa meal (17.6 g/100 g d.m)^19^ and dried beet pulp (9.2 g/100 g d.m.)^20^.

The content of crude oils and fats, as expected for this energy tobacco variety, is high in the inflorescence as well as in the whole plant, but it is reduced in the stem-leaf samples (Table 2). The crude oils and fats content observed for the whole plant fraction is on average higher than what is detectable in both grass and legume plants while for the stem-leaf fraction the contribution of lipid is consistent with what is found in grass plants, such as Italian ryegrass, cocksfoot, perennial ryegrass (*Lolium perenne* L.), corn and sorghum^13,15–17^. According to Hagos *et al*.^21^, high levels of lipids, especially rich in unsaturated long chain fatty acids, can negatively affect the anaerobic digestion of the biomass, leading to problems in digestors such as blocking and microbial inhibition. Similarly, as far as the use of cv. *Solaris* as fodder is concerned, it should be noted that the lipid content of the diet intended for ruminants should not exceed 5% of dry matter consumption to prevent the depression of fibre fermentation^22^. In general, this constraint militates in favour of stem-leaf and whole plant fractions of cv. *Solaris*, as potential fodder.

The range of values observed for the crude ash content varies remarkably among the investigated fractions, inflorescence, whole plant and of stem-leaf (Table 2) suggesting that contamination with the ground may have occurred at the time of samplings. This underlines the necessity to develop a collection technique *ad hoc* for the possible use of the plant as feedstuff. When compared to values reported for standard grass and legume plants, highest ash contents (12–15 g/100 g d.m.) are detectable in ryegrass, cocksfoot, fescue (*Festuca arundinacea* Scrheb.), red and white clover while the alfalfa meal has an average ash content of 11.4 g/100 g d.m. However, higher crude ash content (16–20 g/100 g d.m.) is reported for both forage and biogas biomass of cabbage (*Brassica oleracea* L.), sugar beet (*Beta vulgaris* L.) leaves and necks, and rapeseed (*Brassica napus* L.)^13,15–17^.

The thermo-pluvio- metric data observed in the cultivation period were not significantly related with the organic matter, crude protein, crude oil and fat and crude ash contents of whole plant cv. *Solaris* biomass.

### Nitrogenous components

As a further contribute to knowledge on cv. *Solaris* as feedstock with alternative proteins, the protein KOH soluble content in average accounts for 4.93 and 6.83 g/100 g d.m., for stem-leaf and whole plant respectively (Table 2). On this regard, one of the emerging trends in food science is addressed toward the conversion of non-edible vegetables (plants or waste) into soluble protein isolates to be used in the food industry. In facts, it is known that about 50% of soluble proteins in plant leaves is mainly represented by the enzyme ribulose-1,5-bisphosphate carboxylase (RuBisCo) of great interest in industrial food formulations^23,24^.

Furthermore, considering the protein fraction full potential, Table 3 shows for the first time the average amino acid profile of the cv. *Solaris* fractions and compare them with some values reported in the literature for grass and legume standard plants^15,25^. The whole plant and the stem-leaf fractions of cv. *Solaris* show an amino acidic profile similar to what is reported for standard grass plant for leucine, isoleucine, valine, histidine, glycine and serine, lysine and threonine contents. High levels of arginine are also measured in the whole plant fraction, consistently with values reported for legume plants (Table 3). The amino acid profile of the cv. *Solaris* inflorescence fraction is very close to what reported for alfalfa meal for its content in lysine, threonine, leucine, isoleucine and valine^15^.

**Table 3:** Average content (±s.d.) in amino acid (g/100 g d.m.) of cv. *Solaris*. Values reported in literature for grass and legume plants are shown for comparison^15,25^.

Amino acid	cv. *Solaris* Whole plant	cv. *Solaris* Inflorescence	cv. *Solaris* Stem-Leaf	Grass plant	Legume plant
Lys	0.63 (0.29)	0.83 (0.53)	0.51 (0.05)	0.79	1.29
Met	n.a.	n.a.	n.a.	0.33	0.31
Met + Cys	n.a.	n.a.	n.a.	0.71	0.86
Trp	n.a.	n.a.	n.a.	0.13	0.18
Thr	0.69 (0.21)	0.91 (0.29)	0.57 (0.08)	0.72	10.1
Leu	1.19 (0.35)	1.53 (0.42)	0.99 (0.15)	1.22	1.77
Ile	0.65 (0.14)	0.85 (0.21)	0.52 (0.03)	0.65	1.02
Val	0.84 (0.21)	1.11 (0.27)	0.68 (0.04)	0.55	1.24
His	0.29 (0.08)	0.44 (0.14)	0.23 (0.02)	0.32	0.52
Pro	0.94 (0.98)	1.37 (1.60)	0.42 (0.08)	n.d.	n.d.
Arg	3.74 (0.31)	1.58 (0.14)	0.65 (0.08)	0.85	1.2
Phe	0.72 (0.19)	0.98 (0.28)	0.58 (0.03)	1.33 (Phe + Tyr)	2.06 (Phe + Tyr)
Tyr	0.4 (0.11)	0.56 (0.18)	0.34 (0.12)		
Gly	0.92 (0.30)	1.16 (0.40)	0.73 (0.06)	1.52 (Gly + Ser)	2.11 (Gly + Ser)
Ser	0.69 (0.21)	0.96 (0.33)	0.58 (0.06)		
Ala	0.91 (0.23)	1.13 (0.34)	0.78 (0.07)	n.d.	n.d.
Asp	1.89 (0.95)	2.95 (2.5)	1.18 (0.10)	n.d.	n.d.
Glu	2.06 (0.7)	3.08 (0.78)	1.39 (0.16)	n.d.	n.d.

n.a. = not available; Lys = lysine; Met = methionine; Cys = cysteine; Trp = tryptophan; Thr = threonine; Leu = leucine; Ile = isoleucine; Val = valine; His = histidine; Pro = proline; Glu = glutamic acid; Gly = glycine; Ser = serine, Arg = arginine; Phe = phenylalanine; Tyr = tyrosine; Asp = aspartic acid; Ala = alanine.

These results candidate the cv. *Solaris* biomass as an interesting source of alternative proteins to be used in food preparations.

It should be noted that the leaves of conventional smoking tobacco varieties are not recommended for animal feeding because of the relevant presence of alkaloids and their possible adverse effects on human and animal health^14^ as well as on forage palatability^4^. The content of total alkaloids expressed as nicotine in the investigated samples (Table 2), ranging from 0.09 to 0.70 g 100 g^−1^ d.m., does not seem to represent a constrain for a sustainable use of cv. *Solaris* biomass as forage. According to literature, the nicotine content of dried tobacco leaves ranges in average from 0.3 to 3.5% depending on plant varieties, leaf position, plant maturity at harvesting, practices of cultivation and climate conditions^14^. The nicotine level of cv. *Solaris* should be therefore monitored before its introduction in the diet, in order to avoid risks. In facts, the absence or the possible presence of alkaloids only in trace must be considered a fundamental prerequisite for a potential use of this cultivar as an ingredient into the diet for herbivores. On the other hand, it must be considered that small amounts of many anti-nutritional factors are naturally present in many feedstuffs intended for feeding ruminants, *e.g*. alkaloids in legumes. Moreover, the results from an on-field trial on dairy heifers fed ensiled cv. *Solaris* whole plant showed that growth performances and animal welfare parameters were not affected by the dietary treatment compared to the control group fed a diet containing only meadows hay as forage^26^.

### Cell wall and carbohydrate components, and biochemical methane potential

The average content of Neutral Detergent Fibre (NDF) observed in the investigated fractions of cv. *Solaris* (Table 2) are consistent with values reported for legume plants, especially in red clover, but also in permanent meadow biomass/forages^15^. The Acid Detergent Fibre (ADF) content is consistent with values reported for both grass and legume plants (Table 2) and particularly associates this cultivar to biomass/forages of permanent meadow, cocksfoot, Italian ryegrass, fescue, alfalfa and white clover^13,15–17^. From Table 2 it is also relevant the content of Acid Detergent Lignin (ADL) which is on average much higher in both inflorescence and whole plant samples than in stem-leaf fraction that, on the other hand, is close to what is reported for legume plants, especially in alfalfa and sainfoin. Moreover, the straws are characterized by lignin values of 14 g/100 g d.m.^13,15–17^.

The representation of fibre in cv. *Solaris* is given by NDF, ADF and ADL values that provide essential information on the plant cell wall composition, *i.e*. in terms of hemicellulose (NDF minus ADF), cellulose (ADF minus ADL) and lignin (ADL) contents whose effects on anaerobic ecosystems are very different, as well as a more precise characterization of gastrointestinal fill (NDF) of a forage, main responsible for the voluntary intake in ruminants^13^.

The amount of fibre available for the complex anaerobic ecosystem activities (NDF minus ADL), shown in Table 2, is consistent with values reported in literature for legume plants, being in average equal to 62.3% NDF, 72.6% NDF, and 83.2% NDF, respectively for inflorescence, whole plant and stem-leaf fractions. More in detail, the good available fibre resources in both entire plant and stem-leaf fractions of cv. *Solaris* is close to that found in white clover and sainfoin, even though being characterized by a lower content of cell wall (NDF)^13,15–17^ so that legumes are considered the best forages when the ruminants’ voluntary intake is physiologically low, as it is at the onset of lactation.

The cellulose content of cv. *Solaris* fractions (Table 2) ranged from 19.76 (±2.88) g/100 g d.m. to 28.12 (±5.02) g/100 g d.m. respectively for inflorescence and steam-leaf. Hemicellulose content, instead, was around 8.48 (±4.28) g/100 g d.m., 9.14 (±2.07) g/100 g d.m. and 11.39 (±2.73) g/100 g d.m. respectively for inflorescence, whole plant and stem-leaf (Table 2). These values are in line with what is reported for forages of permanent meadows and alfalfa that are known to be fermented at good rate in the rumen^13^.

Non-Fibrous Carbohydrates (NFC) content, including organic acids, sugars, starch, fructans and all the constituents of the cell wall that are lost during the detergent analysis of fibre^13^, showed high variability in the investigated samples of cv. *Solaris* whole plant (9.26 ± 7.33 g/100 g d.m.) with values consistent with the lowest levels reported in literature for grass plants (10–19.5 g/100 g d.m.)^17^. However, the total sugar content in both whole plant and stem-leaf cv. *Solaris* fractions, variable from 0.42 g/100 g d.m. of stem-leaf and 1.41 g/100 g d.m. of inflorescence, is in average consistent with values observed in fresh grass plants, whereas the starch contents (Table 2), although variable, are consistent with values recovered in alfalfa but lower than what detectable in grass plants and permanent meadows^13,16,17^.

In literature it is reported that high levels of sugars generated during anaerobic fermentations reduce methanogenesis^21^.

From a nutritional standpoint, values reported for whole plant and stem-leaf biomass fractions appear more balanced respect to what has emerged for the inflorescences. The fractions whole plant and stem-leaf of cv. *Solaris* lie at the level of a grass plant of good quality in terms of crude protein and fibrous carbohydrates contents, although presenting higher levels of lignin, close to what is observed in legume plants.

Moreover, the content of sugars and starch suggests that cv. *Solaris* could be conserved by ensiling the biomass harvested 60 days after the seed collection. Ensiled whole plant of cv. *Solaris* was observed to be a good substitute for a portion of hay in dairy heifer diet^26^.

The comparison with compositional data reported in literature for grass and legume plants allowed us to estimate the energy content of whole plant and stem-leaf ranging between 5.33 and 5.83 MJ Net Energy of lactation (NEl)/kg d.m. and 5.48–5.94 MJ Net Energy for weight gain (NEg)/kg d.m.

No significant associations were found between the thermo-pluvio-metric data recorded during the cultivation period and the fibrous components (NDF, ADF and ADL) of whole plant cv. *Solaris* biomass.

When considered cv. *Solaris* as energy feedstock, consistently with the described compositional data, the biochemical methane potential (BMP) tested on whole plant samples by anaerobic digestion showed for the first time quite encouraging results, summarised by an average biogas production of 290 (±75) Nm^3^/t organic matter (or volatile solids) whose 58% is represented by biomethane, as Table 2 shows. Among lignocellulosic crops evaluated for biogas/methane production under BMP operating conditions, *Panicum virgatum* L., *i.e*. switchgrass, is reported to produce 125 Nm^3^ CH_4_/t organic matter^27^ consistently with values observed for grass silage (128–140 Nm^3^ CH_4_/t organic matter), rye ensiled (140 Nm^3^ CH_4_/t organic matter) and gliricidia (G*liricidia sepium*, L) leaves (165–180 Nm^3^ CH_4_/t organic matter) while higher values are reviewed for alfalfa and rapeseed crops (240 Nm^3^ CH_4_/t organic matter)^27^. Much lower biogas values than cv. *Solaris* are reported for rice (*Oryza sativa* L.) straw (74 Nm^3^ biogas/t organic matter), cotton (*Gossypium* L.) stalks and mixed green leaves biomass (180 Nm^3^ biogas/t organic matter)^28^.

These results further extend the potential destinations for this versatile cultivar for the use in animal nutrition and in biomethane production, besides those already studied, *i.e*. source of biofuel^1,3,4^ and protein from the seed cake for piglet feeding^5^.

### Major minerals

Finally, as a further contribute to advanced knowledge on cv. *Solaris* biomass, for the first time the macro-minerals profile is reported in Table 4. It is worth noting the high calcium (Ca) content, about the double of what detectable in legume plants, while the phosphorus (P) content is consistent with what observed especially in sainfoin, alfalfa, corn, barley, fescue, permanent meadows as well as in sugar beet leaves and necks^13,15–17^.

**Table 4:** Average (±s.d.) content (g/100 g d.m.) of minerals measured in samples of cv. *Solaris*. Values reported in literature for grass and legume plants are shown for comparison^15,17^.

	cv. *Solaris* Whole plant	cv. *Solaris* Inflorescence	cv. *Solaris* Stem-Leaf	Grass plant	Legume plant
Ca	2.48 (0.57)	1.35 (0.31)	3.35 (0.94)	0.43 (0.14)	1.30 (0.35)
P	0.27 (0.04)	0.39 (0.03)	0.21 (0.04)	0.31 (0.13)	0.27 (0.07)
Mg	0.62 (0.16)	0.51 (0.11)	0.73 (0.13)	0.15 (0.05)	0.24 (0.10)
K	4.33 (1.28)	3.96 (0.66)	4.13 (0.94)	1.85 (0.57)	2.12 (0.42)
Na	0.0439 (0.0288)	0.0367 (0.0274)	0.0521 (0.0415)	0.05 (0.04)	0.02 (0.01)
Cl	1.52 (0.28)	1.09 (0.14)	1.69 (0.35)	n.a.	n.a.

Ca: Calcium, P: Phosphorus, Mg: Magnesium, K: Potassium, Na: Sodium, Cl: Chloride, n.a.: not available.

Because of the high Ca/P ratio, the eventual use of cv. *Solaris* as forage for ruminants would require attention in balancing the dietary mineral components, while its use in late pregnancy diet would be avoided in ruminants, due to the high risk of metabolic diseases in the *peripartum* phase.

Values higher than what detected in grass and legume plants are indicated also for magnesium (Mg)^13,15–17^. The average content of sodium measured in cv. *Solaris* samples was similar to values reported in literature for grass and legume plants^13,15–17^, while the potassium (K) content shows very high values. Among the factors influencing the K content of forages, also reported high for plants at early vegetative stage (1–4 g 100 g^−1^ d.m.), it has to be mentioned that, besides soil characteristics and environmental conditions, fertilization is one of the major cause of very high K levels of forages^29^. High levels of K in forages are considered etiological factors of diseases in the *peripartum* of the cow so that it is crucial especially in this delicate physiological phase to consider the balance between cations and anions of the diet^30^.

The results of the present study allow to extend the *Nicotiana tabacum* cv. *Solaris* multitasking potential but further investigation on qualitative aspects of analytic components are required for its use in animal nutrition.

Taken together, the results here presented place for the first time cv. *Solaris* as a candidate for a competitive repositioning of innovated tobacco cultivation, especially in the marginal areas traditionally suited to tobacco and devoted to animal breeding. However, the suggested multiple potentialities of cv. *Solaris* raise questions about management of the crop, with reference to timing and harvesting mode, storage techniques and the development of various strategies depending on the destination of products and derivatives.

## Data Availability

The datasets generated during the current study are available from the corresponding author on reasonable request.
